# Actinic keratosis and surrounding skin exhibit changes in corneocyte surface topography and decreased levels of filaggrin degradation products

**DOI:** 10.1111/exd.14089

**Published:** 2020-03-13

**Authors:** Anne J. Keurentjes, Kornelis D. de Witt, Ivone Jakasa, Lars Rüther, Patrick M. J. H. Kemperman, Sanja Kezic, Christoph Riethmüller

**Affiliations:** ^1^ Coronel Institute of Occupational Health Amsterdam UMC, location AMC Amsterdam The Netherlands; ^2^ Laboratory for Analytical Chemistry Department of Chemistry and Biochemistry Faculty of Food Technology and Biotechnology University of Zagreb Zagreb Croatia; ^3^ Dermatest GmbH Münster Germany; ^4^ Department of Dermatology Amsterdam UMC, location AMC Amsterdam The Netherlands; ^5^ Department of Dermatology, Dijklander Ziekenhuis Purmerend The Netherlands; ^6^ Centre for Nanotechnology Serend‐ip GmbH Münster Germany

**Keywords:** actinic keratosis, atomic force microscopy, filaggrin, stratum corneum

## Abstract

Actinic keratosis (AK) is a frequent premalignant skin lesion mainly caused by chronic sun exposure. AK lesions are often surrounded by invisible, subclinical alterations, called field of cancerization (FoC). Definition of FoC is of importance for therapy management; however, the criteria and non‐invasive tools to characterize FoC are lacking. Atomic force microscopy (AFM) proved to be a suitable tool for detection of changes in the corneocyte surface topography in inflammatory skin diseases, which share similar clinical features with AK such as hyper‐ and parakeratosis. Therefore, in this study we applied AFM to investigate AK and surrounding skin obtained by non‐invasive collection of the stratum corneum (SC) with adhesive tapes. Furthermore, we determined degradation products of structural protein filaggrin (natural moisturizing factor, NMF), which previously showed association with the changes in corneocyte surface topography. Ten patients with multiple AK on the face were recruited from the outpatient clinic. SC samples were collected from the AK lesion, skin sites adjacent to the AK, 5 cm from the AK and retroauricular area. Corneocyte surface topography was determined by AFM, and NMF by liquid chromatography. The AK lesion showed alterations of the corneocyte surface topography characterized by an increased number of nanosize protrusions, which gradually decreased with the distance from the lesion. NMF levels show an inverse pattern. Atomic force microscopy showed to be a suitable tool to detect changes in the corneocyte surface topography on the AK lesion and surrounding skin in a non‐invasive manner.

## 1 BACKGROUND

Actinic keratosis (AK) is a common premalignant skin lesion in humans showing dramatic increase, especially in regions with high ultraviolet (UV) exposure and in individuals with light skin type. Due to high prevalence and increasing age, AK poses a substantial burden for the affected individual and the health system. If untreated, AK can progress into squamous cell carcinoma (SCC). Up to 82% of SCCs arise within, in close proximity, or contiguous to AK,^1^ however the factors which predispose development of SCC remain unclear. Often on sun‐exposed skin sites, multiple AKs are present, and the recurrence rate and development of new AK is high, which can be explained by existence of a so‐called field of cancerization (FoC), an area around the AK which shows subclinical alterations.^2^ Therefore, several guidelines^3^, ^4^ recommend treatment should not only be focused on the visible lesion, but also include an entire sun‐exposed field preventing recurrence and development of new AK lesions. In contrast to “lesion‐directed treatments,” including cryotherapy, laser therapy, curettage and dermabrasion, several therapeutic options are available which are “field‐directed” such as retinoids, imiquimod, 5‐fluorouracil, photodynamic therapy, diclofenac and ingenol mebutate.^5^ Definition of field of cancerization area is of great importance for therapy management; however, the criteria and non‐invasive tools to characterize FoC are lacking.

### 1.1 Questions addressed

This study explores the suitability of a morphological biomarker, that is the corneocyte topography to detect alterations in the skin in AK and surrounding area by using a non‐invasive tape stripping technique and atomic force microscopy (AFM).^6^ Additionally, in the tapes, the natural moisturizing factor (NMF) was determined, as it previously showed a close association with alterations in the corneocyte topography.^7^, ^8^

## 2 EXPERIMENTAL DESIGN

### 2.1 Patients

Ten patients (5 females) with Fitzpatrick skin type I or II,^9^ and multiple actinic keratosis lesions on the forehead or on the scalp were recruited at the outpatient clinic in the Dijklander Ziekenhuis (Purmerend, The Netherlands). The study was conducted in accordance with the principles of the Declaration of Helsinki (2013) and was approved by the Ethics Committee of AMC, Amsterdam, The Netherlands. Participation was voluntary, and written informed consent was obtained. The average age was 75.2 years (range 55‐90).

### 2.2 Tape stripping

**Figure 1:**
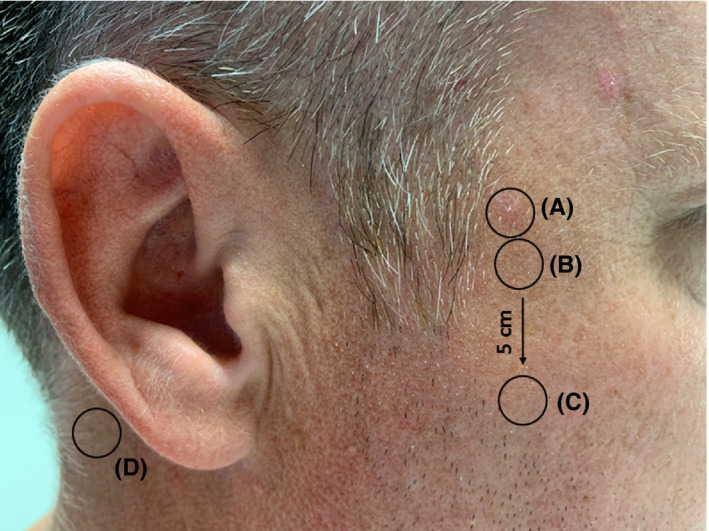
Sampling locations. A, AK lesion, (B) adjacent to AK lesion, (C) at 5 cm distance of AK lesion and (D) retroauricular

Stratum corneum samples were harvested by using adhesive tape strips, a non‐invasive method which is extensively used in experimental studies.^10^, ^11^, ^12^ The tape disc (1.54 cm^2^, D‐Squame; CuDerm Corp.) was attached to each skin site and gently pressed onto the skin for 5 seconds. The tape strips were gently removed with tweezers and placed individually in a cryo‐vial. From each skin site, 9 consecutive tapes were collected. Samples were taken from the following skin sites (Figure 1):

1. The visible actinic keratosis;
2. Next to the border of the redness surrounding the actinic keratosis;
3. A visibly non‐affected site at al least 5 centimetres from the actinic keratosis;
4. Retroauricular.

### 2.3 Atomic force microscopy

Tape strips were glued onto glass slides and subjected to AFM contact imaging with no further preparation as described earlier.^13^ In brief, 10 randomly chosen corneocyte areas of (20 µm)^2^ were subjected to AFM imaging in contact mode (Dimension 3100 apparatus using DNP tips; Digital Instruments, Santa Barbara, USA). Circular nano‐objects of size < 500 nm (CNO) were counted by computer vision algorithms (Serend‐ip GmbH). For details, see Appendix S1 and Figure S1. The average count of 10 areas of (20 µm)^2^ is referred to as Dermal Texture Index (DTI).

### 2.4 Natural moisturizing factor (NMF)

The stratum corneum NMF (sum of histidine, 2‐pyrrolidone 5‐carboxylic acid, trans‐urocanic acid and cis‐urocanic acid) was determined according to the slightly adopted method described in detail elsewhere.^14^

Detailed protocols are provided in Appendix S2.

## 3 RESULTS

**Figure 2:**
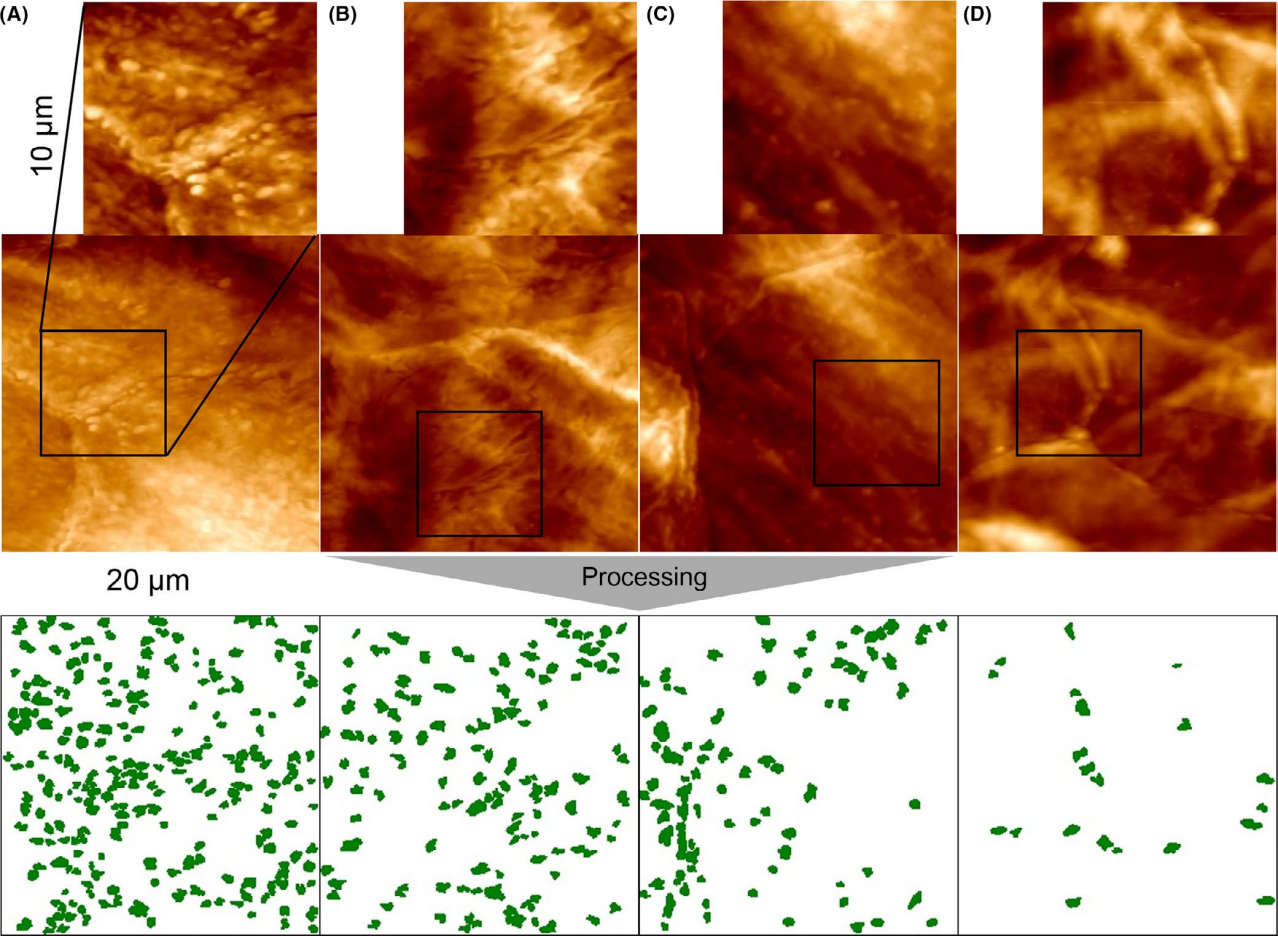
AFM images of (A) AK lesion, (B) skin site adjacent to AK, (C) skin site at 5 cm from AK and (D) retroauricular skin site. Presence of protrusions on the corneocyte surface on the AK and surrounding skin sites (A‐C). Green spots in the lower panel represent circular nano‐objects (CNO) identified by a trained software tool. Number of CNO per (20 µm)^2^ is expressed as DTI

**Figure 3:**
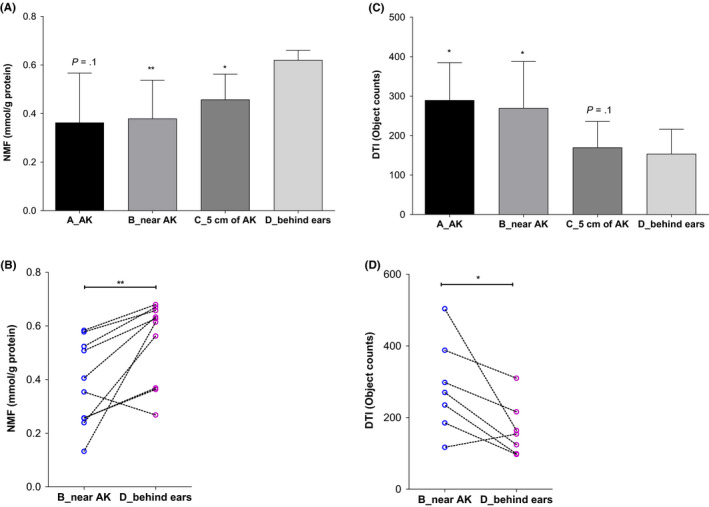
A and C, Levels of NMF and DTI at different skin sites, averaged for 10 subjects. B and D, Individual levels of NMF and DTI at skin sites B (adjacent to AK lesion) and D (retroauricular). Asterisks give the significance level of the difference in NMF or DTI levels between skin sites (A‐C) and D (retroauricular). *P < .05 and **P < .01

The corneocyte surface of the AK (A) shows profound alterations of the surface topography manifested as a dense abundance of circular nanosize objects, CNO (Figure 2). Lesional skin of the AK was devoid of the fibrous meshwork. Also, the skin site adjacent to the AK (B) shows clearly presence of the protruding CNO, which becomes less evident on the skin site at a distance of 5 cm from the AK lesion (C). In contrast, the corneocyte surface collected from the retroauricular skin site showed fairly smooth appearance with fibres (D). These changes in the surface topography are well reflected in the number of CNO per area (DTI, Dermal Texture Index), which shows gradual decrease from the AK to more distant skin sites (Figure 3C). A significant difference in DTI between the control retroauricular skin site (D) was found for the visible AK (A), the adjacent skin site (B) and borderline significant (*P* = .1) for the skin site at 5 cm from AK (C) (Figure 3C and D).

The levels of NMF show an inverse pattern, and the highest NMF levels have been found at retroauricular skin site (D), gradually decreasing towards AK (Figure 3A). A significant difference in NMF levels between the control retroauricular skin site (D) has been found for the skin site adjacent to AK (B), but also for the skin site at a distance of 5 cm from the AK lesion (C) (Figure 3A and B). The NMF levels on the AK lesion were lower than those on the control retroauricular skin, although the difference between these sites did not reach statistical significance (*P* = .1).

## 4 CONCLUSIONS

The findings of this explorative study show that AFM is a suitable technique to detect and quantify alterations in the corneocyte topography, not only in the AK lesions, but also in the adjacent skin areas without visible changes. The number of nanosize protrusions, expressed as DTI, showed a significant decreasing trend with increasing distance from the AK lesion (A). An inverse pattern was found in the NMF levels: the highest NMF level was found at the control retroauricular skin site (D), showing gradual decrease towards the AK lesion (A).

This study revealed for the first time the changes in the corneocyte surface texture and NMF levels in AK. However, abundant presence of nanosize protrusions accompanied by reduction in NMF levels have previously been reported for atopic dermatitis and psoriasis,^7^, ^15^ skin diseases associated with hyper‐ and parakeratosis, which are also the main features of AK.^16^ Increased DTI has also been found in knockout mouse models with alterations in the filaggrin‐NMF pathway.^8^ In that study,^8^ a decrease in DTI was associated with decrease in elastic modulus revealing alterations in the mechanical properties of the corneocytes, likely caused by reduced hydration leading to the shrinkage of the cornified envelope. Interestingly, Berkey et al^17^ showed that skin exposure to UVR caused decrease in the surface cohesion, this effect being reverted after treatment with an emollient or humectant. In concordance with the study of Berkey et al,^17^ showing alterations in mechanical properties of corneocytes after UVR, Engebretsen et al^18^ found elevated DTI levels on the cheek in participants who self‐reported higher exposure to sunlight.

An essential finding is that changes in DTI and NMF levels are not only confined to AK lesions but also to the surrounding areas with apparently normal skin, usually referred to as FoC. Existing subclinical changes can explain the local recurrence, eruption of new AKs or development of SCC in the same area. Therefore, recent European consensus recommendations and expert opinions emphasize the need to treat not only the visible AK lesion, but also the subclinical AK.^4^ While conventional biopsy and histopathological analysis are still regarded as the gold standard for identifying pathophysiological features of AK, this approach is not feasible for larger skin areas.^19^ Therefore, to detect the FoC, various non‐invasive imaging techniques such as dermatoscopy, fluorescence, hyperspectral imaging (HIS),^20^ reflectance confocal microscopy (RCM) and optical coherence tomography (HD‐OCT) have been utilized.^19^ Some limitations of these techniques are a small field of view leading to slow imaging processes and the lack of quantitative outcome. Next to imaging techniques, the AK Field Assessment Scale, based on clinical examination, was developed by Dréno, et al^21^ In this clinical tool, the percentage of the area affected with AK in the face or scalp is graded, as well as the severity of hyperkeratosis and degree of sun damage.

In the present study, we showed that AFM is a promising tool to determine subclinical changes in the skin areas adjacent to the AK. The advantage above visual scoring hyper‐ and parakeratosis, is that AFM provides a quantitative measure of the subclinical actinic changes, which may assist in therapy decisions (lesion‐ or field‐directed), and monitoring of the therapy. The further advantage is that the collection of the samples is simple, fast and non‐invasive. AFM analysis can be performed on a later moment, minimizing the burden for the patient and clinician.

A limitation of this explorative study is the small sample size. Moreover, we did not validate the DTI values with histological data. Further research in a larger patient population, side‐to‐side comparison with other techniques, such as RCM, and monitoring of DTI before and after treatment are therefore warranted. Establishment of a “threshold” for actinic damage might support the decision whether and which skin area should be topically treated.

## CONFLICT OF INTEREST

Christoph Riethmüller holds a patent on nanoanalysis of cell topography EP 2 435 829 B1, US 8,798,935 B2.

## AUTHOR CONTRIBUTIONS

AK, KW, IJ, PK, CR and SK designed and performed the experiments, collected data and statistically analysed data, and drafted the manuscript. CR and LR performed the atomic force microscopy. IJ analysed the samples (NMF). All authors reviewed and approved the manuscript.

## Supporting information


**Appendix S1.** Background
**Appendix S2.** Experimental Design
