# Supplementary material for: Actinic keratosis and surrounding skin exhibit changes in corneocyte surface topography and decreased levels of filaggrin degradation products
Source: Exp Dermatol. 2020 Mar 13;29(5):462–6. doi: 10.1111/exd.14089 (PMC7317372; doi:10.1111/exd.14089)
Supplement: Supplementary file 1 — Appendix S1. Background Appendix S2. Experimental Design [file EXD-29-462-s001.docx]

# **Associated Supplement**

# Appendix S1: Background

##### Atomic Force Microscopy

AFM proved to be an useful tool for analyzing biological samples in their native state (Fig. S1). As a mechanical tool, AFM showed promising results on corneocytes in previous studies on cell stiffness.^[1, 2-5]^  Furthermore, it enables visualization of cell surface structure down to nanometer resolution.^[6, 7]^ AFM generates topography data via the interaction-forces between a probing tip mounted onto a flexible cantilever and the sample surface. Height data recorded by line-wise scanning are used to draw a topographical map with nanometric resolution.^[2, 8]^


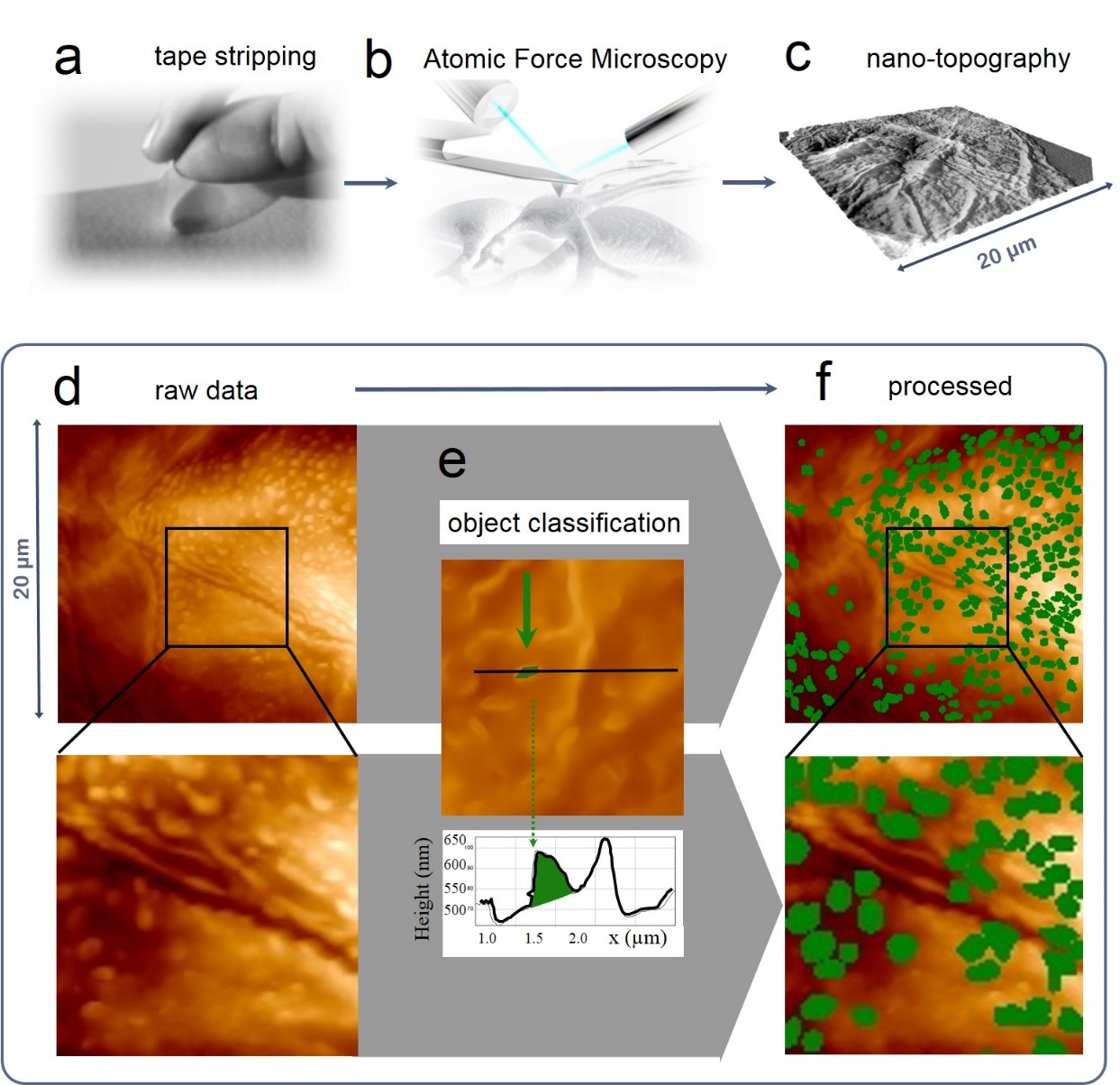


*Fig. S1. Extraction of topographical biomarker DTI from skin samples: corneocytes adhering to tape strips (a) are subjected to analysis by atomic force microscopy (AFM), yielding a topographical map with a height resolution of 1 nm. From the raw data height map (d), computer vision is trained to recognize circular nano-objects (CNO) protruding from the surface (e) and then identifies the number of CNO’s (f). The Dermal Texture Index (DTI) is defined as the average count of CNOs in 10 images of (20µm)2 per sample.*

##### Natural Moisturizing Factor

Natural Moisturizing Factor (NMF) is a pool of several hygroscopic compounds, where the degradation products of a structural protein filaggrin comprise for more than 50% of the total content.^[9]^ NMF has a central role in the regulation of the epidermal barrier (skin hydration), and contributes to the elasticity of the skin likely through interaction with keratin.^[9, 10]^ Recently, in knock-out mouse models of the filaggrin pathway we showed that the NMF-levels are associated with alterations in the corneocyte topography and elastic modulus.^[11]^ Since ultraviolet radiation (UVR) showed to downregulate filaggrin expression,^[12]^ and AKs are common on UVR-exposed skin, an association of NMF levels with actinic damage would be conceivable.

## References

1. C. Riethmüller, M. A. McAleer, S. A. Koppes, R. Abdayem, J. Franz, M. Haftek, L. E. Campbell, S. F. MacCallum, W. H. I. McLean, A. D. Irvine, S. Kezic, *J. Allergy Clin Immunol.* **2015,** *136,* 1573.
2. . Milani, J. Chlasta, R. Abdayem, S. Kezic, M. Haftek, *J Mol Recognit.* **2018;** *31,* 2722.
3. C. Gorzelanny, T. Goerge, E-M, Schnaeker, K. Thomas, T. A. Luger, S. W. Schneider. *Experimental Dermatology.* **2006,** *15,* 387.
4. C. K. M. Fung, K. Seiffert-Sinha, K. W. C. Lai, R. Y. Meng, D. Panyard, J. Zhang, N. Xi, A. A. Sinha, *Nanomedicine.* **2010,** *6,* 191.
5. J. D. Beard, R. H. Guy, S. N. Gordeev, *J. Invest. Dermatol.* **2013,** *133,* 1565.
6. D. J. Müller, Y. F. Dufrêne, *Trends in Cell Biology.* **2011**, *21,* 461.
7. Y. F. Dufrêne, T. Ando, R. Garcia, D. Alsteens, D. Martinez-Martin, A. Engel, C. Gerber, D. J. Müller, *Nat Nanetechnol.* **2017,** *12, 2*95.
8. F. Braet, D. J. Taatjes, *Seminars in Cell & Developmental Biology.* **2018,** *73,* 1.
9. S. Kezic, N. Novak, I. Jakasa, J. M. Jungersted, M. Simon, J. M. Brandner, M. A. Middelkamp-Hup, S. Weidinger. *Frontiers in Bioscience.* **2014,** *19,* 542.
10. A. V. Rawlings, C. R. Harding. *Dermatologic Therapy.* **2004,** *17,* 43.
11. J. P. Thyssen, I. Jakasa, C. Riethmüller, M. P. Schön, A. Braun, M. Haftek, P. G. Fallon, J. Wróblewski, H. Jakubowski, L. Eckhardt, W. Declercq, S. Koppes, K. A. Engebretsen, C. Bonefeld, A. D. Irvine, S. Keita-Alassane, M. Simon, H. Kawasaki, A. Kubo, M. Amagai, T. Matsui, S. Kezic, *J. Investig. Dermatol.* **2019.** *[Epub ahead of print]*
12. S. Simonsen, J. P. Thyssen, S. Heegaard, S. Kezic, S. Skov, *Acta Derm Venereol.* **2017,** *97,* 797.

# Appendix S2: Experimental Design

## Natural moisturizing factor

Briefly, NMF components on the 5^th^ consecutive tape were extracted with 300 µl of Millipore water and subsequently analyzed by high-performance liquid chromatography with UV-detector (HPLC-UV). The NMF concentration was normalized for the SC protein amount, determined with a Pierce Micro BCA protein assay kit (Thermo Fischer Scientific, Rockford, Illinois, USA) to compensate for the variable amount of protein on the SC-tape. The levels of NMF in the SC were expressed as mmol NMF/g protein.

Statistical analyses

Data distribution was assessed with the Shapiro-Wilk normality test. Comparing the difference in the NMF- and DTI levels between skin sites was performed with a two-sided paired Student t-test or Wilcoxon matched-pairs signed-rank test. A P-value of <0.05 was considered to be statistically significant. In the figures, data are presented as mean values ± standard error of the mean (SEM). Statistical analyses were performed with GraphPad Prism 8 software (GraphPad Software, San Diego, California).
